# Guide on Selection of Optimal Motivational Themes for Use in a Clinical Trial Recruiting Black US Adults: Survey Study

**DOI:** 10.2196/75857

**Published:** 2026-03-19

**Authors:** Tamunotonye Harry, Jingyi Cao, Zaib Hussain, Ruth-Alma N Turkson-Ocran, Stephen P Juraschek, Timothy P Lahey, Timothy B Plante, Yuanyuan Feng

**Affiliations:** 1 Department of Computer Science, University of Vermont, 85 South Prospect Street, Burlington, VT, 05405, United States, 1 8026562239; 2 Division of General Medicine, Department of Medicine, Beth Israel Deaconess Medical Center, Harvard Medical School, Boston, MA, United States; 3 Division of Cardiology, Johns Hopkins University School of Medicine, Baltimore, MD, United States; 4 Department of Medicine, Larner College of Medicine, University of Vermont, Burlington, VT, United States

**Keywords:** clinical trials, recruitment, African American, digital advertising, health disparities, cardiovascular health

## Abstract

**Background:**

Black adults in the United States face significant cardiovascular health disparities, which are likely exacerbated by the underrepresentation of Black adults in cardiovascular clinical trials. The Black adult US population has experienced unique historical events, discriminatory practices, and practical obstacles that might contribute to this underrepresentation in clinical trials. An improved understanding of motivations that encourage or discourage participation in cardiovascular clinical trials can lead to more effective clinical trial recruitment and help mitigate these cardiovascular health disparities.

**Objective:**

This study aimed to determine, using an online survey, which motivational themes in clinical trial recruitment advertisements were most effective in encouraging Black adults to participate in a hypertension-focused trial. We also examined how trust in health care and various demographic factors influenced their decision to participate.

**Methods:**

We conducted an online survey with 829 self-identified Black adults in the United States, using a between-subject design to test 4 literature-derived motivational themes in clinical trial recruitment advertisements: (1) contribution to science, (2) helping the community, (3) lowering blood pressure, and (4) access to perks (US $500 worth of groceries or an equivalent cash amount). We assessed advertisement appeal, willingness to participate, and willingness to recommend clinical trial participation to others using cumulative link mixed models.

**Results:**

Demographic factors played a more significant role than motivational themes in predicting advertisement effectiveness. Adults aged 40‐59 years and individuals diagnosed with high blood pressure were more likely to find the advertisements appealing and express willingness to participate. Urban residents engaged more with the advertisements compared to those in suburban or rural areas. Participants with liberal (odds ratio [OR] 1.37, 95% CI 1.01‐1.85; *P*=.04) and conservative (OR 1.62, 95% CI 1.09‐2.40; *P*=.02) political views were more willing to participate in the clinical trial compared to those with moderate views. However, the “lowering my blood pressure” theme was less effective among individuals who distrusted health care institutions (OR 0.40, 95% CI 0.16‐0.97; *P*=.04) and also reduced willingness to recommend the trial (OR 0.36, 95% CI 0.15‐0.85; *P*=.02). In addition, higher trust levels were unexpectedly associated with lower willingness to participate when exposed to this theme (OR 0.41, 95% CI 0.17‐0.98; *P*=.04).

**Conclusions:**

Demographic targeting (age, health status, and geographic location) is more critical than generic motivational messaging in recruiting Black adults to clinical trials. Successful digital health recruitment requires targeting specific demographic segments with tailored messages, as effectiveness varies significantly across sociodemographic factors. The online survey methodology offers researchers a rapid, scalable tool for pretesting recruitment strategies, though it should complement rather than replace community engagement. These insights can help reduce cardiovascular health disparities by improving clinical trial representativeness.

## Introduction

### Background

In the United States, Black adults face poorer cardiovascular health outcomes compared to other groups [1] and are underrepresented in clinical trials [2] that may benefit them. While some large cardiovascular trials have successfully recruited adequate numbers of Black participants through intensive community engagement [3,4], persistent underrepresentation remains a widespread challenge across most trials [2,5,6], particularly outside major academic medical centers. The drivers of the underrepresentation of Black adults in cardiovascular clinical trials are diverse. While the Tuskegee Syphilis Study [7] is often cited as a contributor to medical mistrust among Black adults, research suggests that knowledge of Tuskegee does not universally deter participation and that its impact varies considerably [8,9]. Contemporary factors, such as ongoing experiences with discriminatory practices and physician bias based on race or ethnicity, mental health status, education, income, and sex within the US health care system, play more substantial roles in shaping trust and participation decisions [10,11]. In addition, practical obstacles to participation, such as a lack of information about clinical trials, health insurance, time, and financial constraints, are significant barriers to participation [12,13], contributing to the failure of clinical trials in the United States to achieve their demographic representation goals [2,5,6]. These contemporary, structural barriers often outweigh historical events in determining participation. Despite the federal government’s efforts to ensure inclusion in clinical trials [14], underrepresentation persists, resulting in disparities in health outcomes for minority racial and ethnic groups. Targeted advertisements may improve the recruitment of US Black adults to trials, but it is unclear what advertisement content might be most beneficial.

To support the successful recruitment of Black adults into a community-based participatory trial focused on recruiting Black urban adults (Groceries for Black Residents of Boston to Stop Hypertension [GoFresh]), we conducted an online survey with 829 self-identified Black adults in the United States to evaluate the effectiveness of a flyer-style trial recruitment advertisement with 4 literature-derived motivational themes. The goal of this study is twofold (1) to identify the optimal recruitment motivational themes for recruiting Black adults into the GoFresh trials and (2) to examine other factors (eg, trust in health care systems and demographic characteristics) that may impact Black adults’ intentions to participate in clinical trials. In addition, this study demonstrates how online survey platforms paired with digital advertisement testing can serve as a rapid, cost-effective tool for pretesting recruitment messages before large-scale deployment. This approach allows researchers to iterate on messaging strategies and identify demographic-specific preferences, complementing traditional community engagement approaches. This digital health approach is particularly relevant as clinical trials increasingly use digital channels (eg, social media and online advertisements) to reach diverse populations, yet often lack systematic evidence about which digital messages are most effective for specific audiences.

We seek to answer a key research question (RQ):

- RQ: What motivates Black adults to participate in clinical trials, and how do demographic factors, health status, and trust in health care influence their responses to different recruitment messages?

Based on prior literature, we developed the following hypotheses:

- H1: Different motivational themes in clinical trial advertisements result in different levels of appeal, willingness to participate, and willingness to recommend the trial among Black adults.
- H2: The effectiveness of motivational themes is influenced by an individual’s trust levels in health care systems.
- H3: Demographic characteristics influence responses to clinical trial recruitment through both direct effects on participation interest and by moderating the effectiveness of different motivational themes.

These hypotheses are grounded in prior research demonstrating that (1) message framing affects health behavior [15,16], (2) trust in health care systems significantly influences Black adults’ willingness to participate in research [11,17,18], and (3) demographic characteristics shape health priorities and information processing [19,20].

### Previous Work (Literature Review)

Clinical trials are essential for discovering new prevention, diagnostic, or treatment approaches and improving population health. Without accounting for diversity, resulting interventions may prove ineffective for broader populations, undermining trust and potentially increasing health costs [21,22]. US clinical trials have consistently underrepresented minority groups despite government policies to improve recruitment [23]. Beyond investigator bias [24], possible reasons include lack of awareness, mistrust of the health care system due to experiences with discrimination or bias, and financial and insurance-related obstacles [12,13,24]. A fundamental barrier to Black adults’ participation in clinical trials is that they are often never explicitly asked or invited to participate [25,26]. When Black adults are approached, they are frequently deemed ineligible due to comorbid conditions, which are prevalent in this population due to longstanding health disparities [27]. This structural exclusion, whether through lack of outreach or restrictive eligibility criteria, represents a critical gap that precedes any consideration of motivational messaging.

Current solutions, such as population-specific trials and community engagement, have increased participation from underrepresented groups [28]. However, these are costly in human resources, time-consuming, and risk selection bias, making them unsustainable long-term [28,29]. Most clinical trials rely on traditional recruitment methods such as recruitment by investigators, local doctors, patient databases, printed materials (eg, flyers and posters), and community organizations, with limited data on the performance of digital strategies (eg, websites, social media marketing, and online advertisements) [30]. As a result, clinical trial recruitment recommendations are primarily based on case studies and individual practitioners’ experiences [30,31]. Despite existing research on recruitment strategy effectiveness, there remains a significant gap in data-driven approaches to systematically understand what truly improves clinical trial recruitment.

Big data and web analytics have transformed advertising across industries [32-34], enabling data-driven decision-making. Initial evidence suggests that these approaches can boost participation among underrepresented groups [35,36], yet most US clinical trials fail to leverage these methods to enhance recruitment diversity [37]. Given this opportunity, clinical trial advertising can be tailored, using data-driven methods, to the values and motivations of the target population. Nevertheless, data-driven strategies carry inherent risks of bias when underlying data underrepresent or stereotypically portray minoritized groups [38,39].

The literature [12,19,20] highlights how specific factors in individuals’ lives influence their decision to participate in clinical trials. Recognizing each community’s unique values allows researchers to develop resonant recruitment strategies, improving enrollment and retention [40]. Integrating big data and web analytics into clinical trial recruitment may help identify patterns and preferences among diverse populations. By analyzing these data, researchers can determine which messages and platforms most effectively reach different demographics, leading to more efficient and inclusive recruitment efforts. This study uses a literature-derived approach to develop 4 motivational themes for the GoFresh trials [12,41-43]. These themes reflect both intrinsic motivations (personal values and community impact) [44-46] and extrinsic motivations (health outcomes and financial benefits) [47,48] documented in prior qualitative and quantitative studies of trial recruitment barriers and facilitators.

### Study Aims

This study has 3 primary aims:

- Aim 1: Determine which motivational themes in clinical trial recruitment advertisements are most effective in encouraging Black adults to participate in a cardiovascular health trial, and identify how these preferences vary by demographic characteristics.
- Aim 2: Examine how trust in health care systems moderates Black adults’ responses to different recruitment message themes.
- Aim 3: Demonstrate proof of concept for using an online survey methodology as a rapid, cost-effective tool for pretesting clinical trial recruitment messages before large-scale deployment.

## Methods

### GoFresh Trials

This study is a collaboration with the GoFresh trials. The GoFresh trials (ClinicalTrials.gov nos NCT05121337 [GoFresh] and NCT05393232 [GoFreshRx]) are investigating the impact of home-delivered healthy groceries on blood pressure in Black adults with untreated and treated high blood pressure. In this study, participants are randomly assigned to receive either 12 weeks of Dietary Approaches to Stop Hypertension–patterned [49] grocery delivery with dietitian assistance or self-directed shopping with a monthly stipend over 3 months. After the intervention period, both groups are observed for 9 more months to determine barriers and facilitators for maintaining the Dietary Approaches to Stop Hypertension diet. The ultimate goal of these trials is to improve the cardiovascular health of Black adults living in healthy food priority areas, with and without treated hypertension. Findings from the trials will provide practical insights into effective and scalable strategies to prevent hypertension among Black adults [50].

### Survey Design

**Figure 1.**
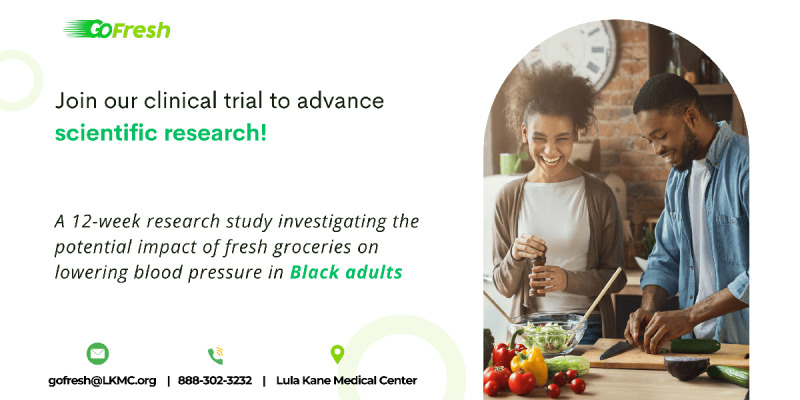
Motivational theme of the advertisement variant: contribution to science.

**Figure 2.**
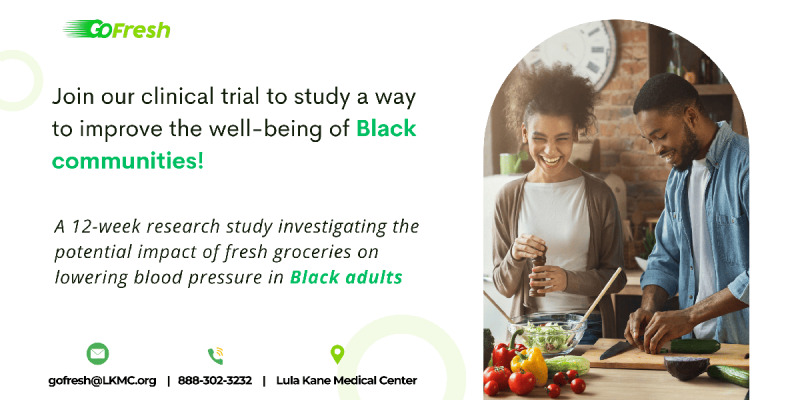
Motivational theme of the advertisement variant: helping the community.

**Figure 3.**
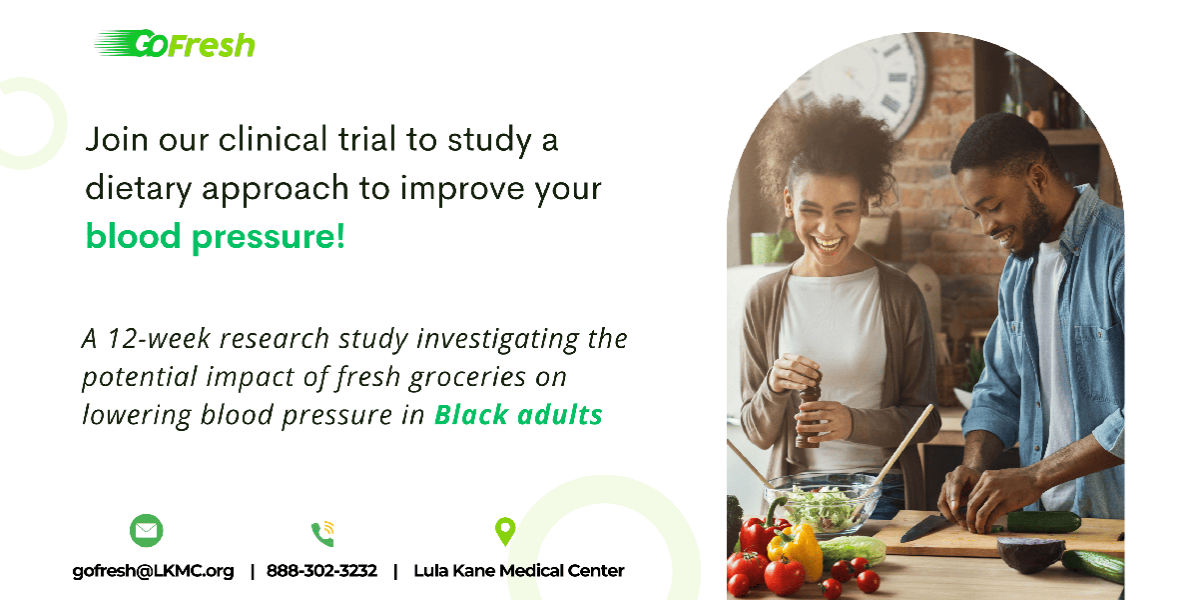
Motivational theme of the advertisement variant: lowering my blood pressure.

**Figure 4.**
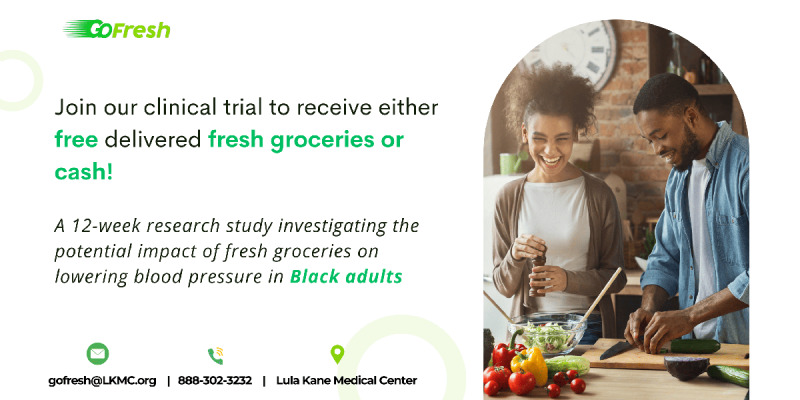
Motivational theme of the advertisement variant: access to perks.

The 4 motivational themes were derived from prior research on factors influencing clinical trial participation [12,41-43]. These themes include contributing to science, helping the community, lowering my blood pressure, and accessing perks (Textbox 1). Flyer-style advertisements were developed for each of these motivational themes (Figures 1-4). The advertisement content and design were reviewed by a community advisory board at Morgan State University to ensure cultural appropriateness and relevance before being tested in the survey. These variations included the GoFresh logo but did not include real contact information for the trial.

Each participant was randomly assigned to 1 of the 4 advertisements and asked to answer the same set of survey questions, rating the level of advertisement appeal, their willingness to participate in the trial, and their willingness to recommend the trial to others. The survey also collected data on participants’ trust in health care systems using a validated scale from Rose et al [51] and Armstrong et al [52], as well as their basic demographic characteristics (refer to Multimedia Appendix 1).

Textbox 1. **List of independent variables.**

**Motivational themes**:

- The 4 motivational themes in the trial recruitment advertisement:

- Contribution to science (science): motivating participants to support scientific research.
- Helping the community (community): encouraging participants to benefit their local community.
- Lowering my blood pressure (blood pressure): promoting personal health improvement.
- Access to perks (perks): highlighting tangible benefits for participation.

**Trust levels in health care**:

- Participants’ trust levels calculated using the Healthcare System Distrust Scale (10-50) [32,33]:

- Distrust (10-30): lowest tertile of trust scores.
- Neutral (31-35): middle tertile of trust scores.
- Trust (36-50): high tertile of trust scores.

**Demographic backgrounds**:

- Potential confounding factors:

- Age group
- Political view
- Gender
- Location (urbanity)

### Study Population

We used the Prolific platform (Prolific Academic Ltd) [53] for web-based participant recruitment. Prolific is an online research platform that connects researchers with high-quality, prescreened participants for surveys and experiments, ensuring ethical standards, fair compensation, and reliable data collection. The platform’s prescreening filter ensured that only individuals who self-identified as Black or African American or mixed race were recruited. Multimedia Appendix 2 shows the inclusion and exclusion criteria used in the GoFresh trials. The survey was deployed on February 2, 2024, and was closed 3 days later. We targeted 1000 respondents. Excluding instances of obvious survey abuse (n=60), participants who provided complete responses were included in the analysis. We did not restrict our sample to individuals with diagnosed hypertension because examining how health status influences recruitment message responses was a key RQ. This also reflects real-world recruitment for cardiovascular trials targeting both prevention and treatment populations.

### Variables and Measurements

#### Dependent Variables

We operationalized the effectiveness of the trial recruitment advertisement as three variables: (1) the appeal of the advertisement—the attractiveness of the advertisement, (2) willingness to participate—readiness to join the advertised clinical trial, and (3) willingness to recommend—likelihood of sharing the clinical trial with others.

The primary goal of advertising appeal is to effectively communicate with a target audience and motivate them to take action. It can be subjective depending on messaging, visuals, or media type (direct mail, social media, print, etc) that are attractive to one’s cognition, emotions, or both, which are rooted in past and present experiences [54]. In clinical trials, an effective trial advertisement motivates (appeals to) the target audience to participate in or recommend the trial within their network.

#### Independent Variables

Participants were exposed to 1 of 4 motivational appeals in recruitment advertisements, each emphasizing a distinct reason for participation: contributing to scientific research (science), helping one’s community (community), improving personal health outcomes, such as lowering blood pressure (blood pressure), or gaining access to tangible benefits (perks). Alongside motivation, trust in the health care system was considered a critical factor in recruitment engagement. Participants’ trust levels were measured using the Healthcare System Distrust Scale [51,52] with scores ranging from 10 to 50. To ensure an even distribution, trust was categorized into 3 levels using tertile cutoffs calculated in RStudio software (Posit PBC) [55]: distrust (10-30), neutral (31-35), and trust (36-50).

These variables provide context for differences in responses to motivational themes and trust levels, helping to refine strategies for trial recruitment. Together, motivation, trust, and demographic characteristics shape participant engagement and the overall effectiveness of recruitment advertisements.

### Statistical Analysis

Sociodemographics and model covariates were tabulated with descriptive statistics. A cumulative link mixed model, a type of multilevel ordered logistic regression model, was used in our analysis. This approach allows estimation of model effects on an ordinal dependent variable while accommodating for random effects (ie, repeated measures). Models were adjusted for demographic factors, including age, political views, gender, and urbanity (location).

#### Main Effect Predictors

We included predictors for advertisement themes, trust levels, and prior diagnosis of high blood pressure by a doctor (high blood pressure status), as well as demographic variables such as age group, income, political view, and gender.

#### Effect Modifier

We used effect modifiers to examine the interactions among independent variables. We evaluated different interaction combinations among important independent variables to address our research hypothesis. After comparing the statistical significance of these interactions, we included interactions between advertisement themes and trust levels (ie, advertisement theme × trust level) to better elucidate how the effectiveness of each advertisement theme varied among people with different levels of trust in health care systems.

#### Predictor Baselines

We set the baseline value for the motivational theme as “contribution to science” since it is the fundamental goal of clinical trials. For other predictors, we selected the neutral or most common values as baselines. For example, for the trust in health care systems predictor, we selected the “Neutral” category as the baseline because it represented the most frequently chosen response within the trust variable. This ensures that the baseline reflects the predominant sentiment among participants, providing a solid reference point for analysis.

#### Data Cleaning

To ensure model convergence, we performed the following steps: (1) converted responses such as “not sure,” “prefer not to answer,” and uncommon responses (eg, gender; “binary”) to the “not available (N/A)” placeholder value, which also made the data more uniform; and (2) combined categories within certain variables, such as trust in the health care system and political views, to simplify and standardize the data for modeling.

#### Final Models

We fit 18 ordered logistic regression models, 6 for each effectiveness metric: appeal, willingness to participate, and willingness to recommend. From these, we selected the best model for each metric (3 models in total) based on a balance between predictor complexity and performance, as measured by the Akaike information criterion score.

#### Results Reporting

For predictors with statistical significance (*P*<.05), we reported the odds ratios (ORs), calculated as the exponential of the model estimate (β), along with the 95% CI. We also calculated the marginal 95% CIs for these interactions to evaluate their statistical significance and range of effect.

Our statistical analyses were conducted using R (version 4.4.0; R Foundation for Statistical Computing) with the ordinal package [56]. No adjustments were made for multiple comparisons.

### Ethical Considerations

This study was approved by the University of Vermont Institutional Review Board (STUDY00002558). All participants provided electronic informed consent before beginning the survey, after being informed about the study’s purpose, procedures, risks, benefits, and their right to withdraw at any time. We anonymized participants’ data before using the remaining 829 survey responses for the final analysis. All data were deidentified, and no personally identifiable information was collected or linked to survey responses. Data were stored securely on password-protected servers accessible only to the research team. Participants who provided complete responses were compensated US $1.60 each, corresponding to an average rate of US $9.58 per hour, through the Prolific platform [53].

## Results

### Analytical Sample

A total of 913 surveys were started, with 897 completed (did not complete: n=16). After removing entries with incomplete fields (n=44), 853 valid responses remained. As shown in Multimedia Appendix 2, we further excluded 24 participants for the following reasons: not identifying as Black, self-reporting a lack of understanding of clinical trials despite being provided with a definition, providing unreasonable or nonrandomized answers, or being age outliers. This resulted in a final analytical sample of 829 responses.

### Analytical Sample Sociodemographics

The sociodemographics of the analytical sample are provided in Multimedia Appendix 3. The 829 valid responses were from participants who self-identified as Black or African American on Prolific; 7 of these participants identified as mixed race. The mean age was 40 (SD 13.0) years. Educational levels varied widely, and most participants (474/829) reported annual incomes between US $35,000 and US $129,999. Participants reporting female and male genders were equally represented (female: 50.2%, 416/829, and male: 49.1%, 407/829). Most participants lived in urban or suburban areas in the United States (large city: 28.2%, 234/829; city: 33.7%, 279/829; and town or suburb: 32.2%, 267/829) and reported liberal (54.6%, 453/829), moderate (26.8%, 222/829), or conservative (18.6%, 154/829) views.

In terms of health status, 29.6% (245/829) reported a prior diagnosis of high blood pressure. The mean score on the Healthcare System Distrust Scale was 32.7 (SD 4.9; range 10-50; distrust [10-30], neutral [31-35], and trust [36-50]). Responses to individual items on the scale showed that 33.4% (277/829) of participants expressed distrust, while 30.3% (251/829) expressed trust in the health care system.

### Appeal of Advertisement

**Figure 5.**
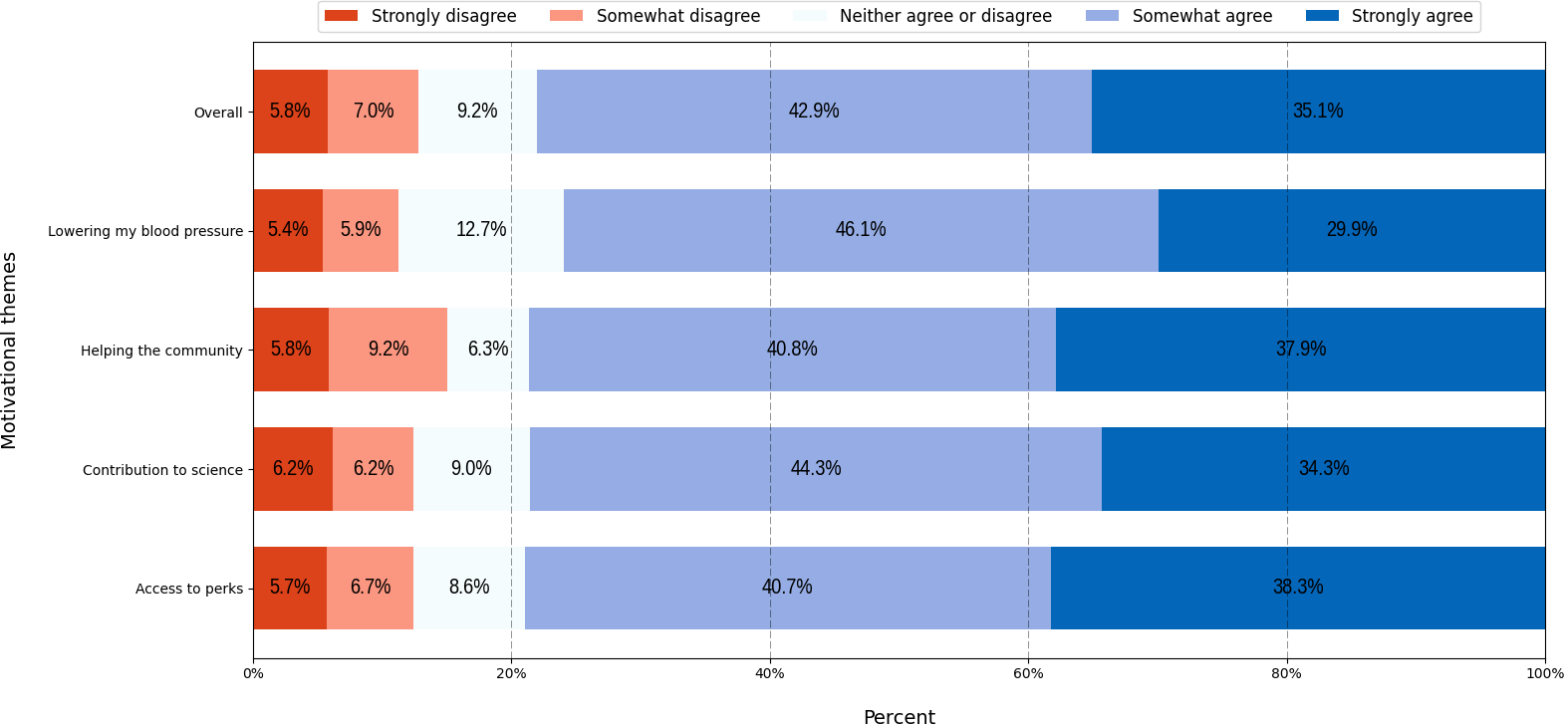
Percentage of participant responses to the appeal of motivational themes in clinical trial advertisements.

As shown in Figure 5, all motivational themes had a high level of appeal, with 76%‐79% (155/204-165/209) of respondents somewhat or strongly agreeing. Notably, the “access to perks” theme was the most appealing, with 79% (165/209) agreeing. In contrast, the “helping the community” theme had the highest level of disagreement, with 15% (31/206) of respondents somewhat or strongly disagreeing.

### Willingness to Participate

**Figure 6.**
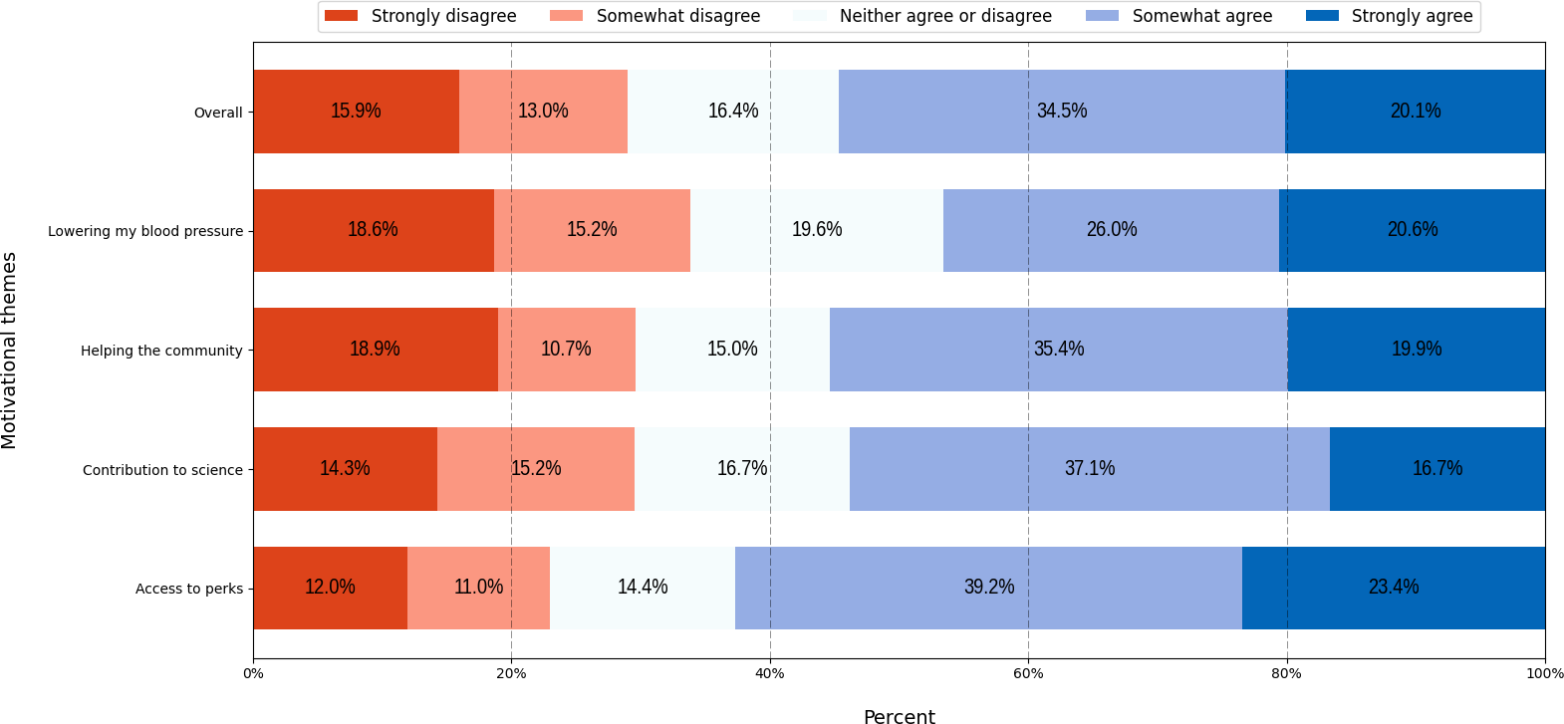
Percentage of participants’ willingness to participate in clinical trials by motivational theme.

As shown in Figure 6, scores for willingness to participate were lower than the appeal of the advertisement, with lower overall agreement to join clinical trials across all 4 motivational themes, ranging from 46.6% to 62.6% (95/204-131/209). The “access to perks” theme yielded the highest willingness to participate at 62.6% (131/209) and the lowest level of disagreement at 23% (48/209). Alternatively, the “lowering my blood pressure” theme had the lowest willingness at 46.6% and the highest level of disagreement, with 33.8% (69/204) strongly or somewhat disagreeing.

### Willingness to Recommend

**Figure 7.**
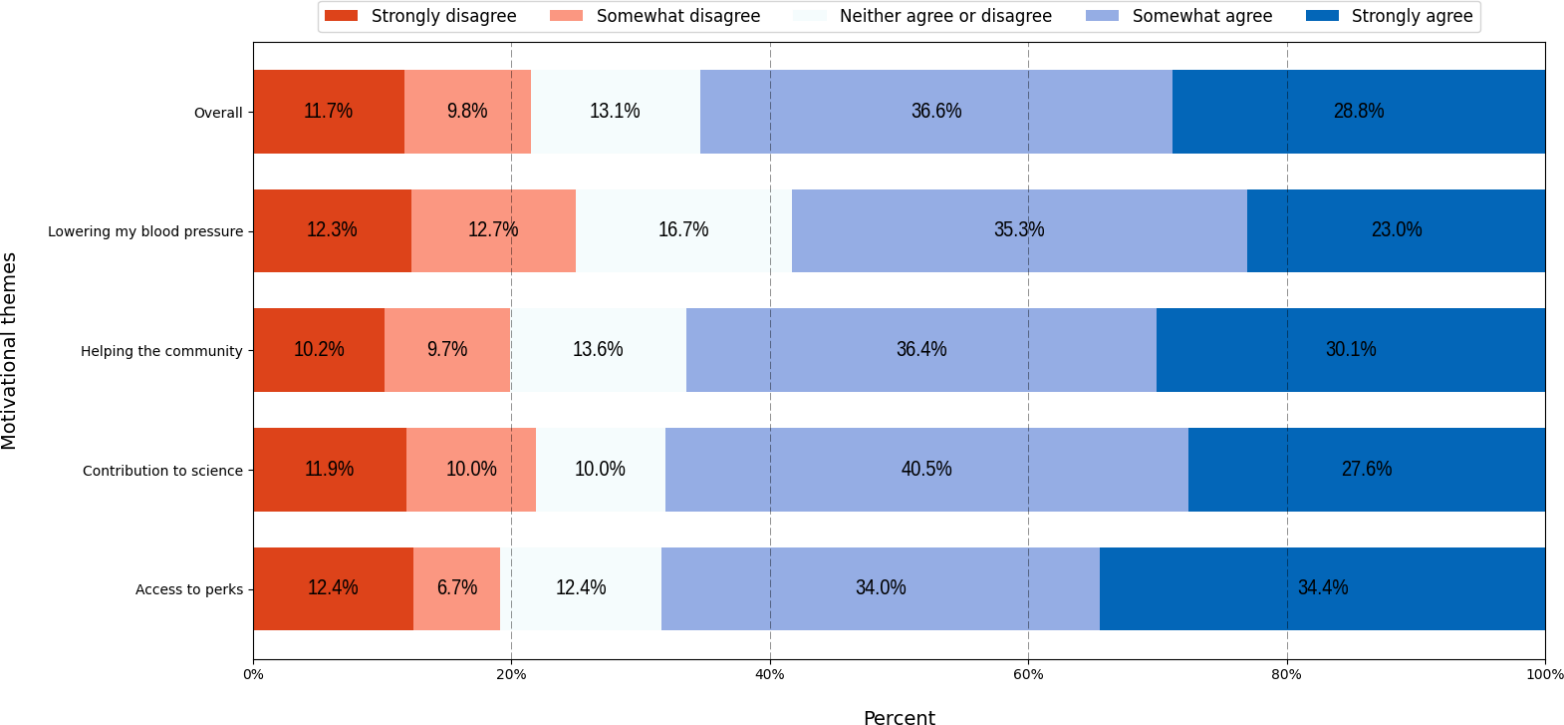
Percentage of participants’ likelihood to recommend clinical trial participation by motivational theme.

As shown in Figure 7, willingness to recommend was generally higher than willingness to participate for most themes, ranging from 58.3% to 68.4% (119/204-143/209) of participants somewhat or strongly agreeing. The highest willingness to recommend was for the “access to perks” theme at 68.4% (143/209). On the other hand, 25% (51/204) of respondents who viewed the “lowering my blood pressure” theme were unwilling (somewhat or strongly disagreed) to recommend the advertisement, the highest level of unwillingness, followed by the “contribution to science” theme at 21.9% (46/210).

### Inferential Statistics

#### Appeal of Advertisement

The model coefficients for the appeal of advertisements are provided in Table 1. Although all groups were compared, certain groups exhibited higher or lower odds of finding a theme appealing.

**Table 1.** Model coefficients for the appeal of motivational themes.

Predictors and effect modifiers	Estimate (β)	Value, OR^a^ (95% CI)	*P* value
Motivational themes			
Research	0	1	—
Blood pressure	0.34	1.41 (0.78-2.54)	.25
Community	0.25	1.29 (0.7-2.37)	.41
Perks	0.05	1.05 (0.57-1.94)	.88
Trust levels			
Neutral	0	1	—
Trust	0.41	1.51 (0.80-2.84)	.21
Distrust	0.52	1.68 (0.90-3.14)	.11
High blood pressure diagnosis			
No	0	1	—
Yes	0.50	1.65 (1.20-2.25)	<.001
Age (years)			
18‐29	0	1	—
30‐39	0.09	1.09 (0.76-1.57)	.64
40‐49	0.42	1.52 (1.01-2.29)	.04
50‐59	0.45	1.57 (1.01-2.43)	.04
60‐69	−0.10	0.91 (0.51-1.61)	.75
>70	0.10	1.1 (0.44-2.73)	.84
Political view			
Moderate	0	1	—
Liberal	0.45	1.56 (1.14-2.13)	.01
Conservative	0.30	1.35 (0.89-2.03)	.15
Sex			
Female	0	1	—
Male	−0.08	0.93 (0.71-1.21)	.58
Location			
Large city	0	1	—
City	−0.2	0.82 (0.58-1.15)	.24
Rural area	−0.2	0.82 (0.45-1.50)	.52
Town or suburb	−0.54	0.58 (0.41-0.83)	<.001
Motivational themes × trust levels			
Blood pressure × trust	−0.87	0.42 (0.17-1.03)	.06
Community × trust	−0.73	0.48 (0.19-1.21)	.12
Perks × trust	−0.15	0.86 (0.34-2.18)	.76
Blood pressure × distrust	−0.92	0.40 (0.16-0.97)	.04
Community × distrust	−0.15	0.86 (0.35-2.11)	.74
Perks × distrust	0.30	1.35 (0.56-3.30)	.51

a OR: odds ratio.

#### Main Effects

None of the motivational themes or trust levels had a significant effect on appeal. However, there were significant associations with appeal among certain groups. Participants who reported being diagnosed with high blood pressure had 65% higher odds of finding the themes appealing compared to those without such a diagnosis (OR 1.65, 95% CI 1.20‐2.25; *P*=.002). People aged 40‐49 years (OR 1.52, 95% CI 1.01‐2.29; *P*=.04) and 50‐59 years (OR 1.57, 95% CI 1.01‐2.43; *P*=.04) had 52% and 57% higher odds, respectively, of finding the themes appealing compared to those aged 18‐29 years. Participants with liberal political views had 56% higher odds of finding the themes appealing (OR 1.56, 95% CI 1.14‐2.13; *P*=.005) compared to those with moderate views. Living in a town or suburb was associated with 42% lower odds of finding the themes appealing compared to those living in large cities (OR 0.58, 95% CI 0.41‐0.83; *P*=.002).

#### Interaction Between Themes and Trust Levels

While the main effects of motivational themes and trust levels were not significantly associated with appeal, their interaction was significant. There was a significant interaction between the “lowering my blood pressure” theme and the “distrust” level (OR 0.40, 95% CI 0.16‐0.97; *P*=.04). This indicates that individuals who viewed the “lowering my blood pressure” theme and expressed distrust in health care systems were 60% less likely to find the clinical trial advertisement appealing. Although this interaction was statistically significant, the broad CI suggests uncertainty regarding the precise magnitude of this effect.

### Willingness to Participate

The model coefficients for willingness to participate are provided in Table 2. Although all groups were compared, certain groups exhibited higher or lower odds of participating in a clinical trial.

**Table 2.** Model coefficients for willingness to participate in clinical trials.

Predictors and effect modifiers	Estimate (β)	Value, OR^a^ (95% CI)	*P* value
Motivational themes			
Research	0	1	—
Blood pressure	0.32	1.38 (0.78‐2.44)	.27
Community	0.16	1.17 (0.65‐2.10)	.60
Perks	0.29	1.34 (0.75‐2.39)	.31
Trust levels			
Neutral	0	1	—
Trust	0.51	1.66 (0.92‐3.00)	.09
Distrust	0.51	1.66 (0.91‐3.04)	.10
High blood pressure diagnosis			
No	0	1	—
Yes	0.63	1.88 (1.39‐2.53)	<.001
Age (years)			
18‐29	0	1	—
30‐39	0.11	1.11 (0.78‐1.59)	.56
40‐49	0.38	1.46 (0.99‐2.16)	.05
50‐59	0.55	1.74 (1.14‐2.65)	.01
60‐69	−0.14	0.87 (0.50‐1.50)	.61
>70	−0.03	0.97 (0.42‐2.24)	.95
Political view			
Moderate	0	1	—
Liberal	0.31	1.37 (1.01‐1.85)	.04
Conservative	0.48	1.62 (1.09‐2.40)	.02
Sex			
Female	0	1	—
Male	0.19	1.21 (0.94‐1.57)	.14
Location			
Large city	0	1	—
City	−0.27	0.77 (0.55‐1.07)	.12
Rural area	0.11	1.11 (0.62‐1.99)	.72
Town or suburb	−0.56	0.57 (0.41‐0.80)	<.001
Motivational themes × trust levels			
Blood pressure × trust	−0.88	0.41 (0.17‐0.98)	.04
Community × trust	−0.46	0.63 (0.26‐1.50)	.30
Perks × trust	−0.13	0.88 (0.37‐2.09)	.77
Blood pressure × distrust	−0.70	0.5 (0.21‐1.17)	.11
Community × distrust	−0.25	0.78 (0.33‐1.85)	.57
Perks × distrust	0.31	1.37 (0.58‐3.20)	.47

a OR: odds ratio.

#### Main Effects

The motivational themes and trust levels did not have a significant effect on willingness to participate. There were significant demographic associations. Participants who reported being diagnosed with high blood pressure (OR 1.88, 95% CI 1.39‐2.53; *P*<.001) had 88% higher odds of being willing to participate in the clinical trial focused on high blood pressure compared to those who had not been diagnosed. Individuals aged 50‐59 years had 74% higher odds of willingness to participate in the clinical trial compared to those aged 18‐29 years (OR 1.74, 95% CI: 1.14‐2.65, *P*=.01). Participants with both liberal (OR 1.37, 95% CI 1.01‐1.85; *P*=.04) and conservative (OR 1.62, 95% CI 1.09‐2.40; *P*=.02) views had 37% and 62% higher odds, respectively, of being willing to participate in the clinical trial focused on high blood pressure compared to those with moderate views. Living in a town or suburb was associated with 43% lower odds of being willing to participate in clinical trials compared to those living in large cities (OR 0.57, 95% CI 0.41‐0.80; *P*=.001).

#### Interaction Between Themes and Trust Levels

The interaction between the “lowering my blood pressure” motivational theme and trust level was found to be significant (OR 0.41, 95% CI 0.17‐0.98; *P*=.04), indicating that individuals who viewed the “lowering my blood pressure” theme and had higher levels of trust were 59% less willing to participate in the clinical trial. Although the interaction was statistically significant, the wide CI indicates uncertainty about the exact size of this effect.

### Willingness to Recommend

The model coefficients for willingness to recommend are provided in Table 3. All groups were compared to each other, but certain groups exhibited higher or lower odds of recommending a clinical trial to others.

**Table 3.** Model coefficients for willingness to recommend clinical trials.

Predictors and effect modifiers	Estimate (β)	Value, OR^a^ (95% CI)	*P* value
Motivational themes			
Research	0	1	—
Blood pressure	0.19	1.21 (0.68‐2.16)	.51
Community	0.18	1.2 (0.66‐2.17)	.56
Perks	0.03	1.03 (0.57‐1.86)	.93
Trust levels			
Neutral	0	1	—
Trust	0.28	1.32 (0.72‐2.42)	.37
Distrust	0.50	1.65 (0.89‐3.06)	.11
High blood pressure diagnosis			
No	0	1	—
Yes	0.31	1.37 (1.01‐1.85)	.04
Age (years)			
18‐29	0	1	—
30‐39	−0.09	0.92 (0.64‐1.32)	.64
40‐49	0.27	1.31 (0.88‐1.95)	.19
50‐59	0.28	1.32 (0.87‐2.01)	.19
60‐69	−0.21	0.81 (0.47‐1.40)	.45
>70	0.44	1.55 (0.64‐3.72)	.33
Political view			
Moderate	0	1	—
Liberal	0.3	1.35 (1.00‐1.83)	.05
Conservative	0.38	1.46 (0.99‐2.16)	.06
Sex			
Female	0	1	—
Male	0.08	1.08 (0.83‐1.41)	.54
Location			
Large city	0	1	—
City	−0.34	0.71 (0.51‐1.00)	.05
Rural area	−0.13	0.88 (0.5‐1.57)	.67
Town or suburb	−0.48	0.62 (0.44‐0.88)	.01
Motivational themes × trust levels			
Blood pressure × trust	−0.56	0.57 (0.24‐1.36)	.20
Community × trust	−0.6	0.55 (0.23 ‐1.33)	.18
Perks × trust	0.3	1.35 (0.55‐3.30)	.52
Blood pressure × distrust	−1.03	0.36 (0.15‐0.85)	.02
Community × distrust	−0.21	0.81 (0.34‐1.95)	.64
Perks × distrust	0.09	1.09 (0.46‐2.61)	.84

a OR: odds ratio.

#### Main Effects

Motivational themes and trust levels did not have a significant effect on willingness to recommend the advertised clinical trial, although there were other demographic associations. A diagnosis of high blood pressure was significantly associated with 37% higher odds of recommending the trial, regardless of the themes (OR 1.37, 95% CI 1.01‐1.85; *P*=.04). Living in a city (OR 0.71, 95% CI 0.51‐1.00; *P*=.05) and in a town or suburban areas (OR 0.62, 95% CI 0.44‐0.88; *P*=.006) was associated with 29% and 38% lower odds of recommending clinical trial advertisements, respectively, compared to living in large cities. While the result for individuals living in a city is statistically significant (*P*=.05), the upper bound of the CI is 1, suggesting the effect could range from a decreased willingness to recommend to no effect.

#### Interaction Between Themes and Trust Levels

The interaction between the “lowering my blood pressure” theme and the “distrust” level was statistically significant (OR 0.36, 95% CI 0.15‐0.85; *P*=.02). This suggests that individuals who viewed the “lowering my blood pressure” theme and expressed distrust in health care systems were 64% less likely to recommend the clinical trial.

## Discussion

While trials such as the Jackson Heart Study [3] and REasons for Geographic And Racial Differences in Stroke study [4] achieved strong Black representation through intensive community engagement, the majority of clinical trials continue to struggle with recruitment diversity [12,13,24]. Our study builds on these successes by providing a scalable methodology for optimizing recruitment messaging across different trial contexts and resource levels.

### Principal Findings

This study makes 3 key contributions to clinical trial recruitment science. First, demographic factors (age, health status, and geographic location) are stronger predictors of Black adults’ interest in cardiovascular trials than motivational message themes themselves, suggesting demographic segmentation should be prioritized in recruitment planning. Second, we demonstrate complex trust dynamics, whereby the “lowering blood pressure” theme (personal health benefit messaging) was less effective among both low-trust and high-trust individuals. Third, we provide proof of concept for using online surveys as a rapid, cost-effective tool for pretesting recruitment messages, offering a scalable methodology that complements community engagement approaches.

Targeting individuals based solely on racial identity overlooks diversity within racial groups, as factors such as socioeconomic status, health conditions, geographic location, age, and political beliefs shape perspectives and motivations to enroll in clinical trials [19,57,58]. Even within the same demographic group, individuals have different needs and motivations. Our research identifies key considerations impacting how Black adults engage with digital clinical trial advertisements: individual-level demographic influences on advertisement effectiveness and trust and information dynamics. Considering these factors can improve design and marketing strategies, increasing the likelihood that recruitment advertisements will resonate with potential participants and enhance the effectiveness of clinical trial recruitment.

### Demographic Influences on Advertisement Effectiveness

Demographic factors such as age, health status, political views, and location significantly influence Black adults’ responses to clinical trial advertisements [19,58,59]. Our findings show hypertension-related trial advertisements resonate most with adults aged 40‐59 years and individuals diagnosed with high blood pressure, suggesting that targeted approaches based on age and relevant health conditions are effective [59]. These individuals, facing age-related health challenges, may actively seek new treatments, increasing their likelihood of participation [44].

While clinical trial advertisements appealed slightly more to Black adults with liberal views, both liberals and conservatives showed willingness to participate. This adds context to how political ideology affects participation, as prior studies [60-62] often overlooked practical considerations driving engagement in underrepresented populations. Our study demonstrates that Black adults’ participation decisions are primarily influenced by pragmatic factors such as incentives, safety, trust, access to treatments, and health assurances, rather than political ideology. This suggests prioritizing tangible benefits during recruitment alongside clear, transparent informed consent.

Black adults living in suburban and rural areas were less likely to engage with clinical trial advertisements compared to those in cities, aligning with literature showing that urban areas have more trial sites and health care facilities. Rural and suburban communities remain underrepresented, have limited trial awareness, face logistical challenges such as transportation barriers, and may harbor mistrust [63-66]. Outreach strategies in these areas should address specific access- and trust-related challenges.

### Trust and Information Dynamics

For clinical trial recruitment, trust fundamentally shapes how potential participants perceive trial motives and their willingness to participate. This manifests through information credibility, transparency regarding risks and benefits, and perceived trustworthiness of sponsoring organizations [67-69]. Our study revealed that trust in the health care system interacted significantly with the “lowering my blood pressure” theme, indicating that for Black adults, trust remains a critical issue influenced by varying motivations and socioeconomic factors, compounded by historical contexts and personal health care experiences [7]. Given these findings, recruitment strategies should prioritize messaging that builds trust with this population.

### Motivational Themes

Black adults indicated reluctance to participate in clinical trials even when they found certain themes appealing. This is reflected in the overall lower levels of agreement in willingness to participate (28.9%) and willingness to recommend (21.5%) compared to the appeal of the themes (12.8%). These results also imply that additional motivators, such as tangible perks, may be necessary to boost participation, as personal health benefit themes such as “lowering my blood pressure” seem less motivating compared to “access to perks,” which appears to be a stronger draw than health benefits alone. Participants also showed a higher willingness to recommend clinical trials with a promise of perks than to participate themselves. This also indicates that participants are more comfortable endorsing or suggesting trials that offer monetary incentives compared to “lowering my blood pressure,” indicating that personal health benefits may not be a popular reason for recommending clinical trials.

Notably, the low willingness to participate or recommend the “lowering my blood pressure” theme may be partly due to the fact that 68.4% (567/829) of participants reported never having been diagnosed with high blood pressure, compared to 29.6% (245/829) who reported having been diagnosed.

We recently published the performance of these themes on a GoFresh clinical trial recruitment website [70]. We found that “lowering my blood pressure,” “access to perks,” and “helping my community” messages had a similar proportion of website visitors who completed the signup form on the GoFresh website. These 3 themes all outperformed the “contribution to science” theme. It is unclear how these themes relate to more relevant outcomes, including clinical trial enrollment and completion. Motivational theme preferences identified in this study might also reflect actual trial enrollment and completion rates. Work to determine these outcomes by theme is ongoing.

### Practical Implications for Researchers

Our findings offer actionable recommendations for clinical trial recruitment:

- Prioritize demographic segmentation: demographic factors (age, health status, and location) are stronger predictors than message themes alone. However, certain themes (eg, “access to perks”) may resonate particularly well with minority populations and should be strategically incorporated when targeting these groups.
- Use online surveys for rapid message testing: this methodology enables cost-effective, rapid pretesting of recruitment messages before investing in full-scale campaigns, allowing iterative refinement based on empirical data.
- Navigate trust dynamics carefully: the relationship between message type and trust varies by community. Among minoritized populations, practical considerations may outweigh health-benefit appeals, particularly when institutional trust is low.
- Combine quantitative and community-engaged approaches: we developed messages with community advisory board input, then quantitatively tested them. This combined approach ensures both cultural relevance and demographic insights.
- Adapt to your specific context: use this methodology as a framework for testing message effectiveness within your own target population, as recruitment patterns are likely context-dependent.

While our study focuses on optimizing motivational messaging, we acknowledge that the most effective recruitment strategy begins with actively and explicitly inviting Black adults to participate. Recruitment messaging, regardless of how well-optimized, cannot address the fundamental barrier of never being asked. Our findings complement, rather than replace, the critical need for proactive outreach and inclusive eligibility criteria that account for the higher prevalence of comorbidities in Black populations.

### Limitations and Future Work

We recognize certain constraints in our study, which we discuss to provide context and ensure a clear interpretation of our results.

This study relied on self-reported intentions rather than actual trial participation behavior. Future research incorporating observed behavioral outcomes could enhance generalizability. Our use of an online panel (Prolific) may limit generalizability to Black adults who lack internet access, are less digitally engaged, or do not participate in online research. The study focused specifically on dietary intervention for blood pressure, and findings may not generalize to other clinical trial types. Future research should explore a broader range of trials involving different medical conditions, interventions, and populations to strengthen applicability. Future work can compare how different audiences respond to messages directly addressing concerns vs neutral messaging to improve communication strategies. We attempted to stratify data by hypertension status, but the skewed distribution (only 245/829, 29.6% observations for the hypertensive group) led to statistical model instability due to sparse data, causing Hessian errors. Simplifying the model would not fully address our RQs, as our goal was to analyze a sample representative of Black adults who might see these advertisements. Future research focused specifically on the hypertensive demographic could examine this group in greater detail, providing more reliable estimates and statistical stability.

A significant limitation is the lack of message variance. We tested 1 message per theme rather than multiple variants. We cannot definitively determine whether effects are attributable to themes or to specific message characteristics. Future research should test multiple message variants per theme to isolate theme-level from message-specific effects [71]. Our quantitative approach risks oversimplifying complex cultural factors that qualitative methods might better capture. Our findings should inform or enhance community-engaged recruitment and not replace it. This methodology served our proof-of-concept goal of rapidly testing recruitment messages before deployment.

While these limitations should be considered, they do not undermine the research validity. The study provides valuable insights that future research can build upon to further enhance the applicability and impact of these findings.

### Conclusion

This study contributes new empirical evidence to the science of inclusive trial recruitment and provides an evidence-based methodology to identify effective advertisements for improving participant diversity. It emphasizes the need for thoughtful design of digital clinical trial advertisements targeting Black adults in the United States. Simply targeting individuals based on racial identity ignores diverse factors such as age, socioeconomic status, location, health conditions, and political beliefs that shape motivations. Effective advertisements must address these differences, offering broadly appealing messages that also meet specific needs. Recruitment messages should be diverse and tailored to address multiple motivations rather than seeking a single, universal driving factor. Addressing trust and cultural sensitivity in recruitment strategies can boost participation, build stronger relationships between health care institutions and communities, and lead to more equitable health research outcomes.

## Supplementary material

10.2196/75857 Multimedia Appendix 1 Survey design.

10.2196/75857 Multimedia Appendix 2 Flowchart of participant inclusion and exclusion criteria for this study.

10.2196/75857 Multimedia Appendix 3 Sociodemographic characteristics of the analytical sample and distribution of motivational themes in clinical trial advertisements by demographic factors.

## Abbreviations

GoFresh: Groceries for Black Residents of Boston to Stop Hypertension
OR: odds ratio
RQ: research question
