# Supplementary material for: Guide on Selection of Optimal Motivational Themes for Use in a Clinical Trial Recruiting Black US Adults: Survey Study
Source: J Med Internet Res. 2026 Mar 19;28:e75857. doi: 10.2196/75857 (PMC13002166; doi:10.2196/75857)
Supplement: Multimedia Appendix 1 [file jmir-v28-e75857-s001.docx]

Url: <https://osf.io/hstpy/?view_only=59fba2bf94384a7c9f75c5d6bc18bab4>
