# Supplementary figures and images for: Guide on Selection of Optimal Motivational Themes for Use in a Clinical Trial Recruiting Black US Adults: Survey Study
Source: J Med Internet Res. 2026 Mar 19;28:e75857. doi: 10.2196/75857 (PMC13002166; doi:10.2196/75857)

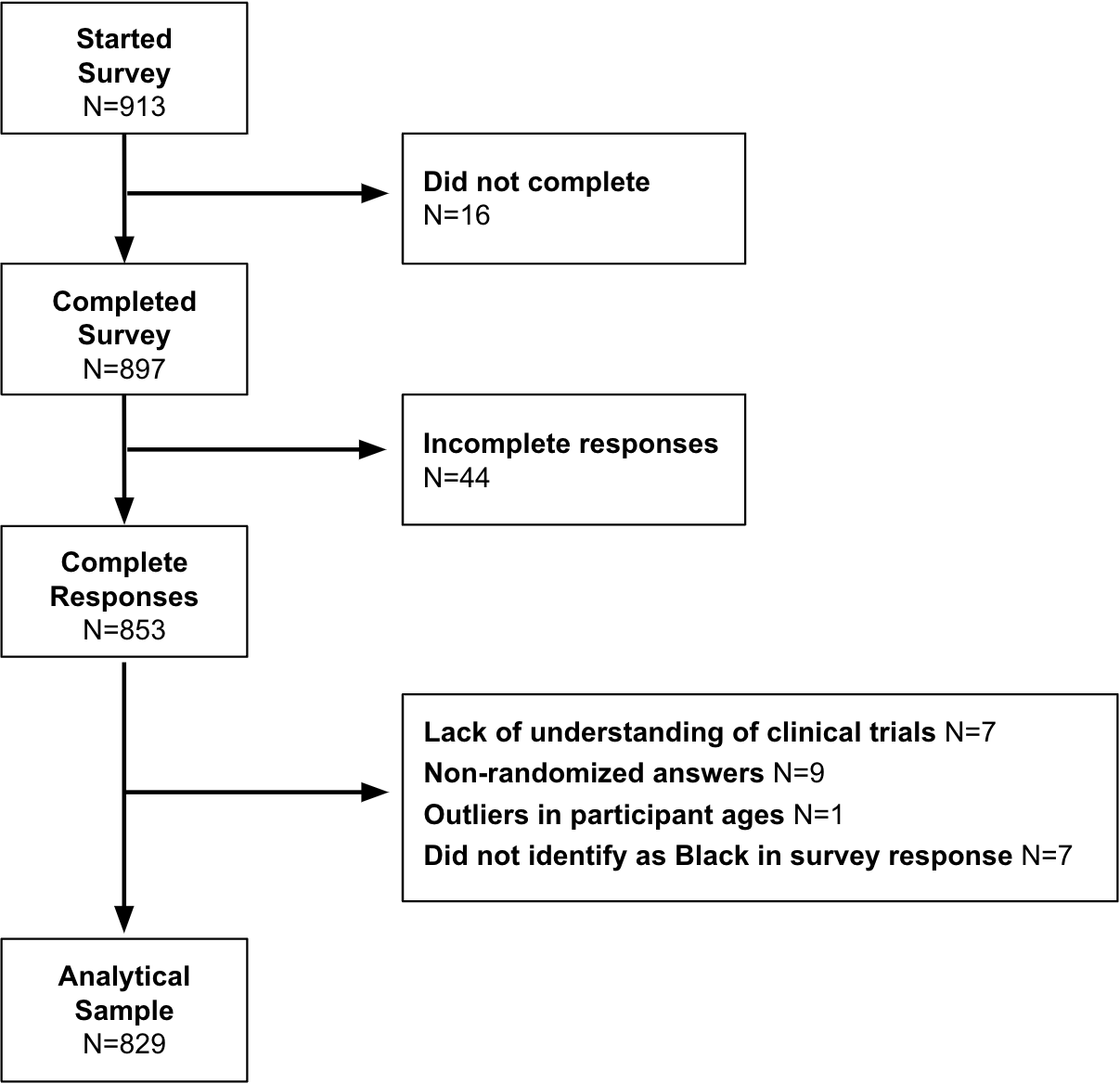

Supplement: Multimedia Appendix 2 [file jmir-v28-e75857-s002.png]
