# Supplementary material for: Guide on Selection of Optimal Motivational Themes for Use in a Clinical Trial Recruiting Black US Adults: Survey Study
Source: J Med Internet Res. 2026 Mar 19;28:e75857. doi: 10.2196/75857 (PMC13002166; doi:10.2196/75857)
Supplement: Multimedia Appendix 3 [file jmir-v28-e75857-s003.docx]

Table A1. Sociodemographics of Analytical Sample.

| **Demographic Variable** | **Count (% of sample)** | **Demographic Variable** | **Count (% of sample)** |
| --- | --- | --- | --- |
| **Age (y)** |  | **Race** | |
| 18 - 29 | 193 (23.3) | Black/African American | 822 (99.2) |
| 30 - 39 | 239 (28.8) | Two or more of the above | 7 (0.8) |
| 40 - 49 | 171 (20.6) |  |  |
| 50 - 59 | 143 (17.2) | **Gender** | |
| 60 - 69 | 64 (7.7) | Female | 416 (50.2) |
| 70+ | 19 (2.3) | Male | 407 (49.1) |
|  |  | Others | 6 (0.7) |
| **Educational Level** | |  |  |
| Less than high school | 3 (0.4) | **Location** | |
| High school or General Education Development (GED) | 77 (9.3) | Large city | 234 (28.2) |
| Associate's degree | 75 (9.0) | City | 279 (33.7) |
| Some college | 170 (20.5) | Town or suburb | 267 (32.2) |
| Bachelor's degree | 319 (38.5) | Rural area | 49 (5.9) |
| Master's degree | 157 (18.9) |  |  |
| More than Master's degree | 28 (3.4) | **Political Views** | |
|  |  | Conservative | 154 (18.6) |
| **Income** | | Liberal | 453 (54.6) |
| Less than $35,000 | 181 (21.8) | Moderate | 222 (26.8) |
| $35,000 - $69,999 | 237 (28.6) |  |  |
| $70,000 - $129,999 | 237 (28.6) | **High Blood Pressure diagnosis** | |
| $130,000 or more | 150 (18.1) | No | 567 (68.4) |
| I prefer not to answer | 24 (2.9) | Yes | 245 (29.6) |
|  |  | I don't Know | 11 (1.3) |
|  |  | I prefer not to answer | 6 (0.7) |
| **Trust Levels** | |  |  |
| Distrust | 277 (33.4) | **Motivational themes** | |
| Neutral | 301 (36.3) | Lowering blood pressure (Blood pressure) | 204 (24.6) |
| Trust | 251 (30.3) | Helping the community (Community) | 206 (24.8) |
|  |  | Access to perks (Perks) | 209 (25.2) |
|  |  | Contribution to Science (Research) | 210 (25.3) |

Table A2. Distribution of motivational themes in the clinical trial advertisements by demographics.

| **Participant Characteristics** | **Motivational themes** | | | |
| --- | --- | --- | --- | --- |
|  | **Lowering blood pressure (Blood pressure)** | **Helping the community (Community)** | **Access to perks (Perks)** | **Contribution to Science (Research)** |
| **Gender** | | | | |
| Female | 107 | 107 | 97 | 105 |
| Male | 96 | 97 | 110 | 104 |
| Others | 1 | 2 | 2 | 1 |
| **Age** | | | | |
| 18-29 | 50 | 42 | 49 | 52 |
| 30-39 | 58 | 64 | 57 | 60 |
| 40 - 49 | 34 | 41 | 51 | 45 |
| 50 - 59 | 44 | 36 | 30 | 33 |
| 60 - 69 | 14 | 16 | 16 | 18 |
| 70+ | 4 | 7 | 6 | 2 |
| **Educational Level** | | | | |
| Less than high school | 0 | 3 | 0 | 0 |
| High school or General Education Development (GED) | 22 | 23 | 14 | 18 |
| Associate's degree | 16 | 20 | 18 | 21 |
| Some college | 39 | 41 | 42 | 48 |
| Bachelor's degree | 78 | 71 | 83 | 87 |
| Master's degree | 40 | 42 | 44 | 31 |
| More than Master's degree | 9 | 6 | 8 | 5 |
| **Income** | | | | |
| Less than $35,000 | 44 | 52 | 39 | 46 |
| $35,000 - $69,999 | 53 | 56 | 54 | 74 |
| $70,000 - $129,999 | 69 | 48 | 68 | 52 |
| $130,000 or more | 34 | 44 | 42 | 30 |
| I prefer not to answer | 4 | 6 | 6 | 8 |
| **High blood pressure diagnosis** | | | | |
| No | 138 | 140 | 150 | 139 |
| Yes | 60 | 61 | 55 | 69 |
| I don’t know | 4 | 3 | 3 | 1 |
| I prefer not to answer | 2 | 2 | 1 | 1 |
| **Political Views** | | | | |
| Conservative | 38 | 45 | 34 | 37 |
| Liberal | 112 | 107 | 119 | 115 |
| Moderate | 54 | 54 | 56 | 58 |
| **Location** | | | | |
| Large city | 60 | 62 | 58 | 54 |
| City | 65 | 74 | 73 | 67 |
| Town or suburb | 73 | 53 | 66 | 75 |
| Rural area | 6 | 17 | 12 | 14 |
